# Remarks on the Medical Statistics of Natchez—a Comparison of Its Mortality While Medicine Was Protected, and Since the Introduction of the “Reformed Practice”

**Published:** 1840-07

**Authors:** Samuel A. Cartwright

**Affiliations:** Natchez


### WESTERN JOURNAL

OF

### MEDICINE AND SURGERY.

JULY, 1840.

### Art. I.—*Remarks on Statistical Medicine, contrasting the result of the empirical with the regular practice of Physic, in Natchez.* By Samuel A. Cartwright, M.D., of Natchez.

I have witnessed two very different eras in the practice of Medicine in Natchez. In the first, the practice was confined to physicians, who had been regularly educated for that purpose, and the doors closed against all species of empiricism. In the second, the practice has not been confined to physicians properly so called, but fully and freely laid open to all kinds of quacks and ignorant pretenders. I am now about to test the results of the practice of medicine in these two eras, standing as I do as a link between them, by the unquestionable facts of statistical medicine.

Statistical medicine or political arithmetic, as it is sometimes called, is that searching department of our science which separates the ore from the dross. It melts down and consumes the scaffolding which elevates empiricism, ephemeral success and accidental popularity, into high places. It undeceives the public by substituting for the caprices of the credulous, the partial and the prejudiced, the unerring results of time and truth. Statistical medicine furnishes the key which opens to public view in a manner the most convincing, simple and summary the actual results of the regular and empirical practice. The doctrine of annuities, successions, reversions and life assurances, are all founded upon the truths afforded by statistical medicine. The facts, which it discloses in regard to the practice of medicine in this city, are well calculated to make every member of this society proud of the profession to which he belongs and of the noble science to which it pertains. They show that the syren voice of the empiric is not to be trusted. They prove that there is no short road to knowledge in medicine—no safety for the afflicted, but in the counsels of those who have patiently climbed the rugged hill of science—no reform to be found by descending lower, but by climbing higher.

The excitement, which of late years, has been kindled throughout the United States against the medical profession under the popular catch word "*reform*," not only struck at physicians, but at some of the most valuable medicines of the materia medica. The medicines, most commonly used by physicians, particularly all the mineral preparations, were denounced as poisons, and pnysicians were represented as a set of men who followed the avocation of poisoning their fellow citizens for mere gain, or from a species of infatuation, and so far from being useful to the public were more mischievous than an equal number of assassins or highway robbers. The excitement was fanned into a flame, with so much zeal, by the manufacturers of nostrums and patent medicines, that many ignorant, but in some instances good meaning persons, were stricken with a species of fanaticism, believing themselves called on to go out into the world to bring about a reform in medicine. Cobblers left their lasts, blacksmiths their anvils, the barber threw aside his shaving-brush, and even grey headed tailors jumped down from the board to become reformers in physic. For some time our strict laws against empiricism kept the reformers out of Natchez. At length, from causes not necessary here to mention, our laws against empiricism were virtually annulled. Very soon afterwards no less than half a dozen reformers, full of zeal, made their way into this city and chose it as the place of their permanent residence to carry out a reformation in medicine. They had never studied the science they came to reform, nor had they ever acquired the elementary education necessary to enable them to begin the study. But their lack of knowledge only made them the louder in denouncing physicians and their remedies. They were particularly hostile to calomel and the lancet. The one, they accused of being in all cases a poison, and the other of being at all times unnecessary and pernicious. They excited the hopes of the afflicted and prevailed on the credulity of the weak, by puffing the many miraculous cures, which, the " reformed system " was said to have effected. But in regard to the more numerous tribes of curable diseases, which the reformed system failed to cure, they put the finger of silence upon their lips. All those cases which baffled the best directed efforts of the regular practitioners, the empirics adduced as witnesses to prove the imperfections and uncertainties of the healing art, thereby endeavoring to shake the confidence of the public in the physicians and their remedies. Many persons who did not become converts to the reformed system, were nevertheless so far influenced by the sophisms, dogmas, and misrepresentations of the empirics, as to lose a large share of their confidence in the regular physicians and failed to make application in due time, for fear of being bled, or having to take calomel or some other drug which the reformers had so repeatedly denounced as poisonous.

I am now about to turn statistical medicine upon the reformers. It is the spear of Ithuriel. The physicians of Natchez in ten years, from the 31st of December, 1823, to the 1st of January, 1834, have lost in all the population, including strangers, 641 patients. In the mean time 337 deaths are recorded not certified by any physician. The whole number of deaths in ten years being 998. This is less than 98 deaths per annum. Estimating the population at an average of 3,000, which falls short, rather than exceeds the true amount, the average mortality in the ten years would be only one in every 30.6-10 per annum. From the facts heretofore contributed to statistical medicine it is known that the average annual mortality of some of the principal cities of the world is much greater than this. According to Dr. Hawkins of the Royal College of physicians, the annual mortality of Naples is 1 in 28; Vienna 1 in 26; Madrid 1 in 29; Rome 1 in 25; Amsterdam 1 in 24. The mortality in all these cities being greater than that of Natchez. According to the same authority the mortality of Paris, Lyons, Strasburg, Barcelona and Nice is from 1 in 31 to 1 in 32, very little less than that of Natchez. In Philadelphia in the year 1832, there were 6699 deaths reported out of a population of 188,397, making the average mortality of that city about 1 in 29.3-10 greater than the average mortality of Natchez. During a period of ten years from 1820 to 1831, the mortality of Philadelphia has varied from 1 in 30.5 to 1 in 42.9. During the same period, the average mortality among fifteen or sixteen thousand negroes in Philadelphia has been 1 in 21.7. According to Dr. Emerson, who has collected much statistical information, Philadelphia is one of the healthiest cities in the United States, and far more healthy than London. Owing to the cupidity of the English government which taxes both the cradle and the grave, the average mortality of Great Britain and the principal cities in it cannot be very correctly ascertained. A large number of births and deaths are not reported, in order to avoid the tax which is levied on all records. The dissenters keep few or no records of births, deaths and marriages. Their records are not received as evidence, consequently the business of registering is monopolized by the parish clerks of the established church. But even with this deficiency, without making any allowance for the unregistered deaths, the average annual mortality of Natchez, during a period of ten years, has been less than the average mortality of London during a period of ten years. The mortality of London from 1790 to 1801 was 1 in 29.3-10. It is somewhat less at present. The average mortality of England and Wales during a period of thirty years, from 1790 to 1821, has varied from 1 in 37 to 1 in 57. In Paris, according to Villerme, the average mortality from the domicil, not including the deaths which occur in the hospitals, (being about one third of the whole,) is one in 58 in those parts of the city inhabited by the wealthy class of citizens, but in the 12th arrondisement which is inhabited by the poorer classes, it is 1 in 24.8-10. Marobale estimates the mortality of France during a period of twelve years from 1817 to 1829, varying from 1 in 27.3-5 to 1 in 53.5. But Napoleon contributed some very valuable and correct information to statistical Medicine. He caused the inhabitants of several departments of France to be accurately enumerated. Officers were appointed with ample powers to make a full and fair registry of all the births and deaths, and to guard against errors from ingress and egress. His object was to learn the natural increase and decrease of the population in order to make it serve as a comparison for the rest of his empire. The departments enumerated contained 2,037,615 individuals—203,102 of whom died in three years—making the annual average mortality in that part of France 1 in 30, being a small fraction greater than the annual average mortality of Natchez for ten years. Thus we find ten or a dozen physicians located in a hot and insalubrious climate, a large portion of the population not being natives or inured to the climate, contending with two yellow fevers, an epidemic cholera, measles, whooping-cough, scarlet fever and small pox; yet under all these disadvantages, the average mortality, during a period of ten years in succession, has been *less* than the average mortality of a large part of France in one of the most salubrious climates in the world—*less* than the mortality in that very portion of France, in which Napoleon had the average correctly ascertained in order to make it a standard of comparison for the rest of his empire—*less* than the mortality in Naples, Madrid, Vienna, Rome and Amsterdam—*less* than in London during a period of ten years—*less* than in Philadelphia during the year 1832, and not much greater than the average mortality in any other town, city or country—no greater, not even as great, if due allowance were made for the deaths which occur among the non-resident population,

More than half the deaths in the Natchez bills of mortality occur among strangers who are not enumerated in the census. If these were subtracted the average mortality of the citizens of Natchez during the whole period of ten years would not exceed 1 in 61 per annum; a less degree of mortality for so long a period than any other town or city in the United States can boast of. I have computed the population of Natchez from 1823 to 1834 at an average of 3000—I believe it however to be more. In 1830 when the population had got to its lowest point, and at a season of the year when the town contained the fewest inhabitants, an actual enumeration, by the officers of the United States government, gave 2789 resident citizens. The bills of mortality in 1830 in a population of 2789 make the average mortality for that year only 1 in 34.8-10. Those acquainted with the history of Natchez know that it gradually decreased from 1823 to 1830, when it slowly increased until 1833, and then in less than twelve months nearly doubled its former population. But to make the lowest enumeration the basis of this calculation, the average mortality, during ten years, would be only 1 in 28.5-10, including the whole of the deaths among strangers, including all still born children, and also including the deaths among from five hundred to a thousand negroes annually brought to this city for sale, prior to the total interdiction of the trade by the legislature of Mississippi in 1836. This would give a less mortality than that of Naples, Vienna, Rome, Amsterdam, Edinburg, Dublin, and at least three fourths of London and Paris, and considerably less than that part of Philadelphia inhabited by negroes.

But how happens it, that the average mortality of Natchez, during a period of ten years, has been so small? How happens it, that while in all other places from 1 to 21 to 1 in 53

of the inhabitants have died annually, in Natchez only 1 in 61 of her citizens, for ten years in succession, has perished annually, and only 1 in 30.6 including strangers and boatmen of which she has always had such great numbers? These astonishing results are not owing to any mistake. All the deaths which occur are faithfully registered by the sexton and reported to the city council. The sexton is bound, under a penalty of fifty dollars for every case of omission, to register and report every death. The population is not estimated too high, because in the year 1830 when the census was taken, by comparing the bills of mortality of that year with the exact population returns, the average mortality is found to be considerably less than the computed average for ten years. The people at a distance, hearing of so many epidemics in Natchez, naturally supposed that the mortality must be very great, and that human life was less secure than in almost any other place. But facts of the most indisputable kind prove that Natchez, in ten years, under the most discouraging and disadvantageous circumstances, lost a fewer number of her citizens by death, in proportion to her population, than almost any other town or city in the civilized world. The reason of so gratifying a result is as plain as the noonday sun. Natchez, during the ten years mentioned, *protected and encouraged science, and science protected and guarded her citizens.* The citizens of Natchez, during these ten years, were protected by strict laws against murderous quacks and empiricism of all kinds. The whole population had confidence in the medical profession, and owing to this confidence, *made timely application for medical assistance,* whenever they found themselves afflicted with any malady whatever. Other towns and cities may have had as good physicians, but the influence of empirics, the ignorance of the people, and their want of confidence in the healing art, besides the great numbers, in Europe, who perish *for the want of bread* and the comforts and necessaries of life, have co-operated to make the healing power and beneficial influences of the medical profession less diffused, felt, and appreciated. But in Natchez, situated in the most favored spot of the most favored land, where plenty and abundance abound, where every inhabitant, bond or free, rich or poor, young or old, has all the solid comforts and necessaries of life at all times at command, the benefits and blessings of the science of medicine were dispersed and diffused throughout her whole population, until the subtle wiles of crafty and designing empirics and pretended reformers, impaired the confidence of the public in the regular medical profession. Leaning with confidence on the arm of science, the citizens of Natchez passed through two yellow fevers, an epidemic cholera, whooping-cough, measles, scarlet fever and small pox, besides the other diseases incident to the climate, and in ten years a fewer number of them perished, in proportion to the population, than in any other town or city from which we have any authentic results. The good effects of confidence in the medical profession, and the benefits to be derived from timely application for medical advice, were clearly demonstrated during the epidemic cholera, which visited this city in May and June of 1833. While in Paris, and many other cities, the ignorant inhabitants, goaded and worked upon by designing empirics, and the venders and fabricators of nostrums, and secret medicines, were accusing the physicians of having poisoned them, consequently refusing and disdaining medical aid and dying by thousands, the good people of Natchez were every where seeking timely medical assistance. The consequence was, that the epidemic, out of the whole population of Natchez in two months, May and June, including strangers, and negroes brought here for sale, only carried off forty-three individuals. Whereas nearly this number died in a single day in many villages and towns of no greater population, wherever the inhabitants abandoned their physicians and looked to empiricism and to nostrums for safety.

I have thus attempted to show Natchez trusting in and wisely protecting science during the long period of ten years—through all this long lapse of years science being triumphant, amidst circumstances calculated to desolate and depopulate any other city. But it is now my task to review the picture and to exhibit Natchez during a period of four years and nine months—a period, with the exception of three months in 1837, remarkable for its health and entire exemption from epidemic diseases. I will present her to the reader—not leaning upon the arm of science—not confiding exclusively to it—not trusting in it—not protecting it by wise legislation, but letting it go, to follow after ignorant, presuming and fanatical empirics, and seeking safety in the patent nostrums of ignorance and fraud. Before the termination of 1833 the laws of Mississippi, which protected the science of Medicine and guarded the people against ignorant presumers and pretended reformers, were virtually annulled. By the first of January 1834 a host of empirics had made their way into our city, and commenced in good earnest, what they called a reformation in medicine. They first began their operations by using every artifice to destroy the confidence of the public, in the virtue of those remedies and means, which the accumulated experience of ages, has found to be the most effectual in the treatment of a large class of diseases—particularly such as occur in warm climates. They used great and unwearied exertions, not only to prejudice the public against most of the medicines which physicians employed, calling them poisons, but they endeavored to destroy public confidence in the physicians themselves and to bring contempt and disrepute upon the regular exercise of the medical art. So great was their zeal, they succeeded in weakening public confidence in the medical profession in a greater degree than could have been expected in so intelligent a community. Some good citizens of Natchez and its vicinity they entirely alienated from it. In some instances they even succeeded in turning those, who owed their lives to the scientific practice of medicine, altogether against it. The natural effect of debility and old age were artfully attributed to the influence of calomel and the lancet. Deaths, which no human means could avert, and which must occur while man is mortal, were said to have been occasioned by poisonous drugs. The more numerous cases, which *got sound and well* under the use of the *very same* drugs, were overlooked. Nothing was likewise said of the many remediable cases which proved fatal under the empirical practice—but the welkin was made to ring with every case which got well under it, or which recovered in spite of it.

I now come to apply statistical medicine to that portion of time which has elapsed, since half a dozen or more empirics have located themselves in Natchez to carry out, what they call, "a reform in medicine." In other words, I am now about to test the pretended reformation in medicine by the unerring results of time and truth.

But first I propose to follow up the practice of the regular physicians. A considerable part of the population, generally embracing the more intelligent citizens, were not seduced by the empirics, but continued to employ as formerly, the regular physicians. During the last four years and nine months, omitting the deaths which occurred during the period of the epidemic of 1837, the regular physicians lost 523 patients.

Admitting that the population of Natchez, since the 1st of January 1834, has been double what it was during the ten years preceding 1834, the number of patients which have died under the care of physicians, during a period of four years and six months, would, if the population has actually doubled itself, be 566; provided their practice was neither more nor less successful than the practice of the physicians of the ten years preceding 1834. If the epidemic be excluded, fewer persons have died since the 1st of January 1834, under the treatment of regular physicians, than did previously. But if the epidemic of 1837 be included in the estimate, 84 more persons have died under the regular physicians, in the last four years and nine months, than the proportional number of deaths which occurred in the practice of the physicians who preceded them. Thus, if the physicians, during a period of ten years in a population of 3000, lost 641 patients, how many, in the same ratio, would die in a population of 6000 in four years and nine months? The relative proportion would be 609. But 693 is the number reported. But in the ten years from 1823 to 1834, besides the 641 patients reported by physicians, there are 337 unreported cases, or cases reported by persons not belonging to the profession. These 337 unreported cases were partly composed of strangers brought here in a dying state from New Orleans; partly of accidental deaths; partly of still born children; partly of intemperate and houseless strangers; partly of persons who refused medical advice, and partly of omissions in the attending physicians to report all the deaths which occurred in their practice. If a population of 3000, in ten years, gives 337 unreported cases, how many unreported cases would a population of 6000 give in four years and nine months. The number would be 320. But the actual number of unreported cases of the last four years and nine months, as proved by the sexton's books, is 622. If 320 be taken from 622 there will remain 302. Therefore, 302 individuals in the last four years and nine months, have either been directly sacrificed on the smoking altars of steam, or had their confidence in the medical profession so much impaired by designing empirics as to forego the advantages of science, and die of remediable diseases. Lest it be supposed that the great excess of unreported cases occurred during the epidemic of 1837, I have consulted the register kept by the sexton, and find that the whole number of unreported deaths, during August, September and October 1837, embracing the whole period of the epidemic, is only 70. By deducting 70 from 302 there remain 232 more unreported deaths in four years and six months (omitting three months for the epidemic,) than occurred during the whole period of the preceding ten years. The total number of deaths, which occurred in ten years, from 1823 to 1834, is 978. If therefore a population of 3000, in ten years, lose 978, how many deaths would occur in a population of 6000 in four years and nine months? The total number should be 929. But the actual number of deaths recorded in the sexton's books, the last four years and nine months, since the pretended reformation in medicine commenced, is 1315. The reformation, therefore, has reformed, in four years and nine months, no less than 386 individuals out of existence. But to put the matter in a plainer light—the average annual mortality of Natchez since empirics have divided the practice with the regular physicians, is no less than one death per annum in every 21.7-10 of the inhabitants: but to estimate the mortality on the actual population of 1837, when Natchez contained its highest population, 6000, it would be, 1 in 22.2; but in the ten years preceding this mixture of empiricism with the regular practice only one in every 30.6-10 died per annum. During these ten years, the number who died under the care of physicians was about 1 in 47 per annum, and the number of unreported deaths during the same period was 1 in 89 per annum. But during the last four years and nine months, including the late epidemic, and including all the cases from the Natchez hospital, the physicians lost 1 in 41 per annum, whereas the unreported deaths of the same period were 1 in 45.7-10 per annum; nearly double the unreported cases of the former period, and exceeding the whole mortality occurring in the practice of the physicians during ten years. But it may be supposed, that the increased mortality of the last four years and nine months, may be owing in part to the greater number of persons who have settled in Natchez, and who have had to undergo that peculiar change called acclimation. It may also be supposed, that the comparatively small mortality of the ten preceding years might be owing to a greater proportional number of citizens leaving town, during the sickly months in the first period of ten years, than in the second period of four years and nine months. Plausible as these suppositions may be to account for the great difference of mortality during the two periods, a voice from the cemetery proclaims, that such is not the true reason. It is well known, that the fever of acclimation, or the stranger's fever, as it is sometimes called, never occurs in the winter or spring. It is likewise known, that no persons fly from town in order to avoid sickness in January, February and March. But what does the voice from the burial ground say? It says, that the total number of dead bodies conveyed there, during a period of ten years, from 1823 to 1834, in the months of January, February and March, was only 159; and that the number carried thither, the last five years in the same months, January, February and March, is no less than 239. Half the time and twice the population ought to give the same results, as twice the time and half the population. If, therefore, the introduction of empiricism, and patent medicines into Natchez had added nothing to the mortality of the place, the aggregate number of deaths which occurred during the first quarter of each of the last six years, should not have exceeded the aggregate number of deaths which occurred during the first quarter of each of the ten preceding years. But they do exceed them, as far as 239 exceed 159. The difference, between these two numbers, gives the precise increase of mortality, during the months of January, February and March, since half a dozen or more deluded empirics made this city their place of residence and commenced, what they called, a reformation in medicine. The average annual mortality, taking these months as the standard, during ten years, was only 1 in 47. But no sooner did illiterate mountebanks make a lodgement in Natchez, than the mortality of January, February and March increased to 1 in 31.4-10 per annum. In the month of April, during the ten years between 1833 and 1834, the physicians lost 39 patients, and during the same time 11 deaths were unreported. The whole mortality for ten Aprils in succession was only 50. But since quackery has been let loose upon the people of Natchez, 85 persons have died during the last five Aprils—being 35 more than the proportional number of deaths that occurred before the empirics proffered their services to the good people of this city.

In other words, making April the standard, the average mortality would be only 1 in 50 per annum of the total population, including strangers, during a period of ten years. But since Natchez has been overrun by empirics, the mortality of the last five Aprils has increased to 1 in 29. 4-10 per annum.

The mortality which occurred in the practice of the physicians in each month of April for ten years, between 1823 and 1834, has been at the rate of 1 in 64. 2-10. The mortality which has occurred in the practice of the physicians of the last five Aprils has been at the rate of 1 in 65. 7-10—nearly equal as far as the physicians are concerned in both periods. Yet in the first period of ten years only 1 in 50 died, but as soon as quackery was introduced, 1 in 29. 4-10 died. It is worthy of particular remark, that the number of deaths reported by the physicians in both periods, not only in the month of April, but every other month of the year, is relatively very nearly the same. Thus, 39 deaths were reported by physicians in the ten Aprils of the first perod, and 38 in the five Aprils of the second period. But while the unreported deaths of the first period were only 11, the unreported deaths of the second period amount to 47. Again, during the first ten of the last fifteen Mays, Natchez lost in all 73 individuals; 40 reported by physicians and 33 unreported cases. But during the last five Mays, she has lost 99 individuals, 33 reported by physicians and 66 unreported cases, or cases reported by persons who are not regular physicians. During the last period the unreported deaths, or those which have been reported by persons not belonging to the medical profession, double the number of deaths reported by physicians; whereas before the introduction of quackery, a large majority of all the deaths were reported by the physicians. To sum up the matter, therefore—in January, February, March, April and May of the ten years preceding 1834, 293 persons died in Natchez; while in the same months for the last five years, 421 have died. In half the time, and twice the population, the number should have been the same. No less, therefore, than 128 individuals have unnecessarily perished, or have been wantonly sacrified, since the first of January, 1834, during the five most healthy months in the year. Taking these five healthy months, as being the months best calculated to test the success of the empirical practice, as the months when empiricism is most popular, (diseases being the mildest, and the principle of life the strongest,) 128 individuals have died, over and above the number which did die in twice the time and half the population, before the empirical practice was introduced, and before public confidence was shaken in the medical profession. If these healthy months were made the standard of comparison, Natchez would lose 331 individuals in five years over and above the number she lost, in ten years and half the population, under the regular exercise of the medical profession, unmixed with quackery. The empirics will no doubt try to evade the issue by disowning the unreported cases, or may even lay them to the charge of the physicians. But the main and essential fact, *that the mortality of Natchez has greatly increased since they came to town and commenced a pretended reformation in medicine, will still stand in bold relief against them.* If they could prove that the physicians neglected to report all the deaths which occurred in their practice, the physicians, on their part, could prove that many of the cases they did report, had previously been subjected to steam, or that the sick had eked out the first period of their disease in the use of nostrums and quack medicines. The medical statistics of Natchez prove in the clearest and most undeniable manner, that previously to the introduction of quackery the annual average mortality for ten years in succession, embracing a very sickly period, rife with epidemic diseases, was much less, or at least very little, if any greater, than the average mortality of most other towns and cities in the world which are reputed healthy. But since the introduction of quackery, the annual average mortality has greatly increased. To charge the increased mortality to the present regular physicians would avail empiricism nothing, unless it could blot out from the records of the city the diminished mortality under the regular physicians, before it was permitted to obtrude itself among them. The present physicians, and those of a former day drank at the same well of science. When the empirics came to Natchez the annual average mortality, including strangers, still-born children, and the deaths occurring among the negroes brought here for sale, was only 1 in 30. 6—and had been at this rate for the ten preceding years, while the mortality among the citizens proper did not exceed 1 in 61; but the very month in which the empirics commenced, in good earnest, to reform a science they had never studied, the mortality suddenly increased to one death in every 21 inhabitants per annum, notwithstanding that Natchez had, in the mean time, ceased to be a market for the sale of negroes, which formerly added very considerably to the bills of mortality, not only from the deaths which occurred among them, but likewise from the contagious diseases, as measles and whooping cough, which they almost annually introduced into the city.

The important fact, that the mortality of Natchez has greatly increased since the introduction of quackery, cannot be evaded by saying, that I have rated the population too high at one period and too low at another. The population in 1830 was known to be 2789. The number of deaths which occurred in that year is likewise known to be 80—which would make the average for that year only 1 in 34. 8-10. The population in 1837 amounted to 6160. The number of deaths which occurred in nine months of that year, omitting entirely the deaths which occurred during the period of the epidemic in August, September and October, amounts to 186—this would give an average mortality of 1 in 24. 4-10 per annum. But if the population since the empirics have been permitted to practise medicine in Natchez, be put at the highest enumeration, and at the lowest enumeration previously to their introduction, still would it be found that the mortality of the city, since the introduction of quackery, has increased in a manner sufficient to demonstrate that the city has gained nothing by the pretended reformation in medicine, but has lost much. I do not say, nor do I believe, that the empirics have actually carried a sufficient number of patients through a full course of steam and No. 6, to account for the great increase of mortality; but I do say, and believe, that the war they have waged against the known and well-tried remedies of the Materia Medica, and the measures they have taken to destroy public confidence in the medical profession, are nearly sufficient of themselves to account for the increased mortality, without taking into the estimate those who died under their scalding treatment.

I owe to the empirics, *en masse*, a public exposition of my opinions in regard to the "reformed system," for having wantonly and wilfully spread, far and wide, a report which was most industriously circulated, that I had, in a good degree, become a convert to their doctrines. This, like many other reports, I viewed as a trick in their trade of deceiving the public. It is true I have used red pepper and lobelia in my practice, but I have used these articles of the Materia Medica in my practice before I ever heard of steam doctors or botanical reformers. If the fanatic Thompson had taken a fancy to calomel and the lancet, instead of red pepper and lobelia, I should not have thrown aside calomel and the lancet for fear of being classed among his disciples by employing the remedies which he and they abused.

The empiric is known by his nostrums and secret combinations, and by his ignorant and indiscriminate use and application of the known and established articles of the Materia Medica. It is not the medicine, but the man, who ignorantly uses the medicine, which constitutes empiricism. No one medicine can be proper in all cases of disease. The empiric treats all cases, however different, very nearly alike, with the same articles, whether vegetable or mineral substances, whether calomel or red pepper and lobelia; but the physician draws his remedial agents from the animal, vegetable and mineral kingdoms, he knows, or should know, the properties and virtues of all; consequently in the treatment of the various diseases, he makes such selections of medicinal agents, for every individual case, as the lights of science, conjoined with his own and the experience of former ages and other countries, may, in his judgment, after a careful examination of the case, seem to be the most appropriate.

In the collection and publication of the medical statistics of this city, my first object was to bring about some action, at home, which might prevent any further and unnecessary sacrifice of human life among my neighbors and fellow-citizens. But I have supposed, that a full and free inquiry in regard to this important matter by the profession in other quarters, might be attended with happy results, and I trust that my essay will be followed by other expositions on the same subject, which will at length open the eyes of the community to the extent and enormity of this evil. It may not be in the power of the profession to induce legislatures to enact laws for the suppression of empiricism; but it is their duty to inform the public as to the evils of quackery. These statistics, it is not in the power of empirics, or of those who countenance them, to gainsay or resist; and if they be extended by physicians in other places where the "*reforming system*" has been in operation, a mass of testimony will be collected which, in the end, must exercise a controlling influence upon public sentiment. It is by the exhibition of such facts as have been presented in this paper, that the judgments of men are to be convinced.

